# Acceptability, feasibility, and individual preferences of blood-based HIV self-testing in a population-based sample of adolescents in Kisangani, Democratic Republic of the Congo

**DOI:** 10.1371/journal.pone.0218795

**Published:** 2019-07-01

**Authors:** Serge Tonen-Wolyec, Salomon Batina-Agasa, Jérémie Muwonga, Ralph-Sydney Mboumba Bouassa, Charles Kayembe Tshilumba, Laurent Bélec

**Affiliations:** 1 Ecole Doctorale Régionale D’Afrique Centrale en Infectiologie Tropicale, Franceville, Gabon; 2 Faculté de Médecine, Université de Bunia, Bunia, Democratic Republic of the Congo; 3 Faculté de Médecine et de Pharmacie, Université de Kisangani, Kisangani, Democratic Republic of the Congo; 4 Laboratoire National de Référence du Sida et des IST, Kinshasa, Democratic Republic of the Congo; 5 Programme National de lutte Contre le VIH/SIDA et les IST, Kinshasa, Democratic Republic of the Congo; 6 Laboratoire de virologie, Hôpital Européen Georges Pompidou, and Université Paris Descartes, Paris Sorbonne Cité, Paris, France; University of California, UNITED STATES

## Abstract

**Background:**

Adolescents living in sub-Saharan Africa constitute a vulnerable population at significant risk of HIV infection. This study aims to evaluate the acceptability, feasibility, and accuracy of home-based, supervised HIV self-testing (HIVST) as well as their predictors among adolescents living in Kisangani, Democratic Republic of the Congo (DRC).

**Methods:**

A cross-sectional, door-to-door survey using a blood-based HIV self-test and a peer-based supervised HIVST approach was conducted from July to August 2018 in Kisangani, DRC. The acceptability and feasibility of HIVST were assessed among adolescents’ consenting to use and interpret HIV self-test. The accuracy of HIVST was estimated by the sensibility and specificity of adolescent-interpreted HIV self-test. Factors associated with acceptability and feasibility of HIVST were analyzed with logistic regression.

**Results:**

A total of 628 adolescents (including 369 [58.8%] females) aged between 15 and 19 years were enrolled. Acceptability of HIVST was high (95.1%); 96.1% of participants correctly used the self-test, and 65.2% asked for verbal instructions. The majority of adolescents (93.5%) correctly interpreted their self-test results. The Cohen’s κ coefficient between the results read by adolescents and by supervisors was 0.62. The correct interpretation decreased significantly when adolescents had no formal education or attended primary school as compared to those currently attending university (37.0% *versus* 100%; adjusted OR: 0.01 [95% CI: 0.004–0.03]). In the hands of adolescents at home, the sensitivity of the Exacto Test HIV Self-test was estimated at 100%, while its specificity was 96.0%. The majority of participants (68.0%) affirmed that post-test counseling was essential, and that face-to-face counseling (78.9%) was greatly preferred.

**Conclusions:**

Home-based, supervised HIVST using a blood-based self-test and peer-based approach can be used with a high degree of acceptability and feasibility by adolescents living in Kisangani, DRC. Misinterpretation of test results is challenging to obtaining good feasibility of HIVST among adolescents with poor educational level. Face-to-face post-test counseling seems to be preferred among Kisangani’s adolescents.

## Introduction

Adolescents aged between 10 and 19 years [1] constitute a vulnerable population at significant risk of HIV infection, particularly girls [2]. Nearly 90% of the world’s HIV-positive adolescents live in sub-Saharan Africa [2]. Approximately 170 young adolescents aged 15–19 years become infected with HIV every day in West and Central Africa, i.e. 62, 000 new infections in 2017 (43,000 among females and 19,000 among males) [3].

Despite the need for HIV testing among adolescents, coverage and uptake seemingly remain poor [1]. Thus, less than 20% of adolescents (15–19 years of age) living in West and Central Africa are aware of their HIV status [4]. The promotion of HIV testing in adolescents requires accepted and effective strategies [5], including HIV self-testing (HIVST), which has been recommended by the WHO as a additional approach to HIV testing services since 2016 [5,6]. Few studies have focused on the acceptability, feasibility, and accuracy of HIVST among adolescents living in sub-Saharan Africa [7–9]. Some existing pilot studies reported high acceptability of HIVST among adolescents in South Africa (75%), Mozambique (85%), and Zambia (86%), especially among those not currently accessing existing HIV testing services [7–9]. Difficulties correctly interpreting invalid or negative test results have been reported among adolescents with a low educational level in Mozambique and Zambia [8,10]. Thus, WHO recommends that the directly assisted HIVST should be implemented among adolescents [5].

In the Democratic Republic of the Congo (DRC), the largest French-speaking country in Central Africa, HIV infection is characterized by its heterogeneous prevalence throughout the country estimated to 1.2%, with adolescents constituting 23% of the population but carrying 14% of the burden of the HIV epidemic [10–12]. In 2016, according to the report of the UNAIDS ALL IN initiative, more than 90% of adolescents are unaware of their HIV status in DRC [11]. Girls are two times more likely than boys to be infected with HIV [3]. HIVST has been recommended by the national program against HIV and AIDS since 2016 [12]. Generally good practicability of HIVST has been already reported among the general adult population living in the DRC [13] as well as among female sex workers living in Kisangani [14]. Yet, according to the DRC’s Strategic Sector Plan for Health 2018–2021 [10], HIVST is also recommended for adolescents aged between 15 and 19 years. This study aimed to evaluate the acceptability, feasibility, and accuracy of home-based, supervised HIVST as well as their predictors and to measure individual HIV testing preferences among adolescents living in the DRC.

## Material and methods

### Study design

This study consisted of a cross-sectional, door-to-door survey using a blood-based HIV self-test (Exacto Test HIV Self-test, Biosynex, Strasbourg, France) [6] and a peer-based supervised HIVST approach. The Exacto Test HIV Self-test (Biosynex) is an adaptation of the finger-stick whole-blood HIV self-test of the professional CE IVD, lateral flow, immunochromatographic HIV rapid test Exacto PRO Test HIV, which has been re-packaged for individual use (with the inclusion of all components necessary in the HIV self-test: disinfectant wipe, compression swab, lancet, sampler stick and dressing) [13,15]. The original instructions for use (IFU) of the Exacto Test HIV Self-test were adapted into a simplified but comprehensive version for the Congolese general public, with pictures showing African people carrying out the test. Ultimately, the pictorial and simplified instructions for use of the HIV self-test comprised an easy-to-read leaflet in French, Lingala and Swahili (the most widely used languages in Kisangani), in A3 format color printing, as described previously [13].

### Study setting

The study was conducted from July to August 2018 in Kisangani, the capital city of Tshopo province and the third-largest urbanized city in the DRC [16], comprising 1.6 million inhabitants, 20% of whom are adolescents, in a context of HIV seroprevalence of 2.3% in individuals aged 15 to 49 years [10]. The city of Kisangani is composed of six townships (Lubunga, Makiso, Kisangani, Tshopo, Kabondo, and Mangobo). Makiso, Tshopo, Kabondo, and Mangobo were arbitrarily included in the survey because of their high population density, good cadastral subdivision, and inclusion of health facilities offering HIV counseling and testing services. The *Cliniques Universitaires de Kisangani*, the *Hôpital Général de Référence de Kabondo*, and the health centers of Neema and Saint Joseph were selected for confirmatory HIV testing, counseling, and care.

### Recruitment of participants

The survey targeted adolescents during the vacation period. All participants volunteered to take part in the study. The inclusion criteria were as follows: ignorance of own HIV status, willingness to be tested for HIV infection, being between 15 and 19 years of age, ability to speak and read the French, Lingala, or Swahili languages, and signed informed consent (parents’ or guardians’ consent for minors [individual under the age of 18]).

Individuals who were already aware of their seropositivity for HIV, who were away from home during the visiting time of the study team, who were unable to communicate in French, Lingala, or Swahili, or who did not provide consent to participate were excluded from the study.

### Sampling and sample size

Random sampling in clusters at three levels was used to select survey participants. The first level corresponded to the different neighborhoods in each of the four townships arbitrarily included in the study, using probability proportional to size sampling. After calculating the demographic weight of each commune and determining the size of the corresponding sub-sample, the neighborhoods (level 1 cluster) in each commune were identified and enumerated. In each neighborhood, 10 streets (level 2 cluster) were randomly selected. On each street, the number, *n*, of households (level 3 cluster), corresponding to *n* = *N*/10, was randomly selected, where *N* was the sample size of the corresponding neighborhood. The number of households to be visited per cluster was determined in proportion to the size of the cluster. Thus, eligible adolescents who were identified at home were enrolled from the selected households at the time of the study visit by the peer educators and counsellors. The population of adolescents in Kisangani was estimated to be 300,000, with a sample size calculated at a 95% confidence level, 0.5 standard deviations, and a 5% confidence interval was estimated at 384 participants.

### Peer educators

In the study, peer educators were adolescents who conducted home-based enrollment of participants to eliminate psychological and social barriers [17,18]. The peer educators fulfilled the following criteria: (i) aged between 18 and 19 years; (ii) willing to participate full time during the study period; (iii) completed at least college or technical school; (iv) fluent in French, Swahili, or Lingala; (v) capacity for interpersonal communication; (vi) available and accessible at any time by telephone. Peer educators were retained on the basis of a favorable evaluation by at least three members of the study team, comprising the coordinator and two counselors (health care workers). Peer educators were trained for one week in HIV testing, including HIVST, pre-test and post-test counseling, HIV prevention, HIV care, practical use of the Exacto Test HIV Self-test, and study objectives. HIV prevention training was solely theoretical and was based on the fact sheets and training modules of the national HIV and STI control program in the DRC [12]. Items concerning HIVST included an overview of HIVST, approaches for HIVST, and the HIVST package.

The respondent-driven sampling method was used to recruit peer educators. A total of 80 peer educators were originally recruited. After evaluation using a post-training semi-structured questionnaire, considered successful when at least 80% of answers were correct, 36 peer educators were ultimately retained. Note that, the peer educators were also recruited during the vacation period; their participation was voluntary; and only their restoration and transportation costs were taken care of.

### Study outcomes

The main study outcomes were the acceptability and feasibility of HIVST. Acceptability was defined as consenting to and using the provided self-test in the participants’ homes. The feasibility of HIVST was evaluated according to adolescents’ ability to use the finger-stick whole-blood self-test autonomously or with verbal instructionsuntil obtaining a valid test result, and to correctly interpret the HIV self-test result in a home-based, supervised setting.

The secondary outcome included adolescents’ preferences for HIV testing strategy (HIVST *versus* voluntary counseling and testing [VCT]), willingness to pay for HIVST, and counseling (pre-test or post-test), as previously mentioned [19]. The willingness to recommend HIVST to a third person and to secondarily distribute the HIV self-test to a third person were evaluated as well.

Following WHO requirements for pre-qualification [20], the field sensibility/specificity and the concordance of adolescent-interpreted HIV self-test results compared to supervisors-interpreted HIV self-test result were estimated to evaluate the accuracy of the Exacto Test HIV self-test in the hand of adolescents. The positive predictive values (PPV) and negative predictive values (NPV) were estimated as well.

### Study procedures

During the home visits, two or three trained peer educators were supervised by one health care worker (nurse) considered as counselor. Eligible members of the same household were offered the option of individual or group pre-test counseling. Thus, a peer educator provided the standard HIV information about testing in one sitting, after which the participants were asked to give written consent to be tested. A 10-minute, face-to-face demonstration of how to use the self-test using the simplified IFU was carried out by one of the peer educators to familiarize adolescents to HIVST process. After completion of the pre-testing phase questionnaire, participants were offered the choice to perform the self-test. In a private setting away from their families and supervised by one of peer educators, participants who accepted HIVST received a self-test kit for performance and interpretation of the results. Participants were then asked to conduct the self-test by themselves. Participants who were identified as having a positive result from the self-test during the survey were advised to seek further evaluation and were referred to HIV care in one of the four selected health facilities according to their preference. Individuals who tested negative for HIV received HIV prevention counseling by counselors. The counselors also encouraged participants to disclose their HIV status to their sexual partners and/or family members, if they felt comfortable in doing so. Finally, participants were invited to complete the exit questionnaire.

### Data collection

A face-to-face, paper-based, semi-structured questionnaire (in the chosen language: French, Lingala, or Swahili) to measure participant socio-demographic characteristics, HIV testing history, knowledge of available HIVST approach, self-reported sexual behavior, HIV risk perceptions, and pre-test acceptability and preference for HIVST was completed by adolescents before performing the self-test. Exit questionnaire measured the acceptability and preference for HIVST in the post-testing phase. All data on feasibility were independently recorded on a standardized sheets by both the participants, the peer educators, and the counselors. The standardized sheet included the information on the blood presence in the square well of the test device, the presence of a line visible adjacent to the letter C, and the interpretation of test result. Note that the peer educators received rigorous training on how to talk to participants who requested verbal instructionsand how to record the information on the observation sheet.

### Reference HIV testing

**Fig 1:**
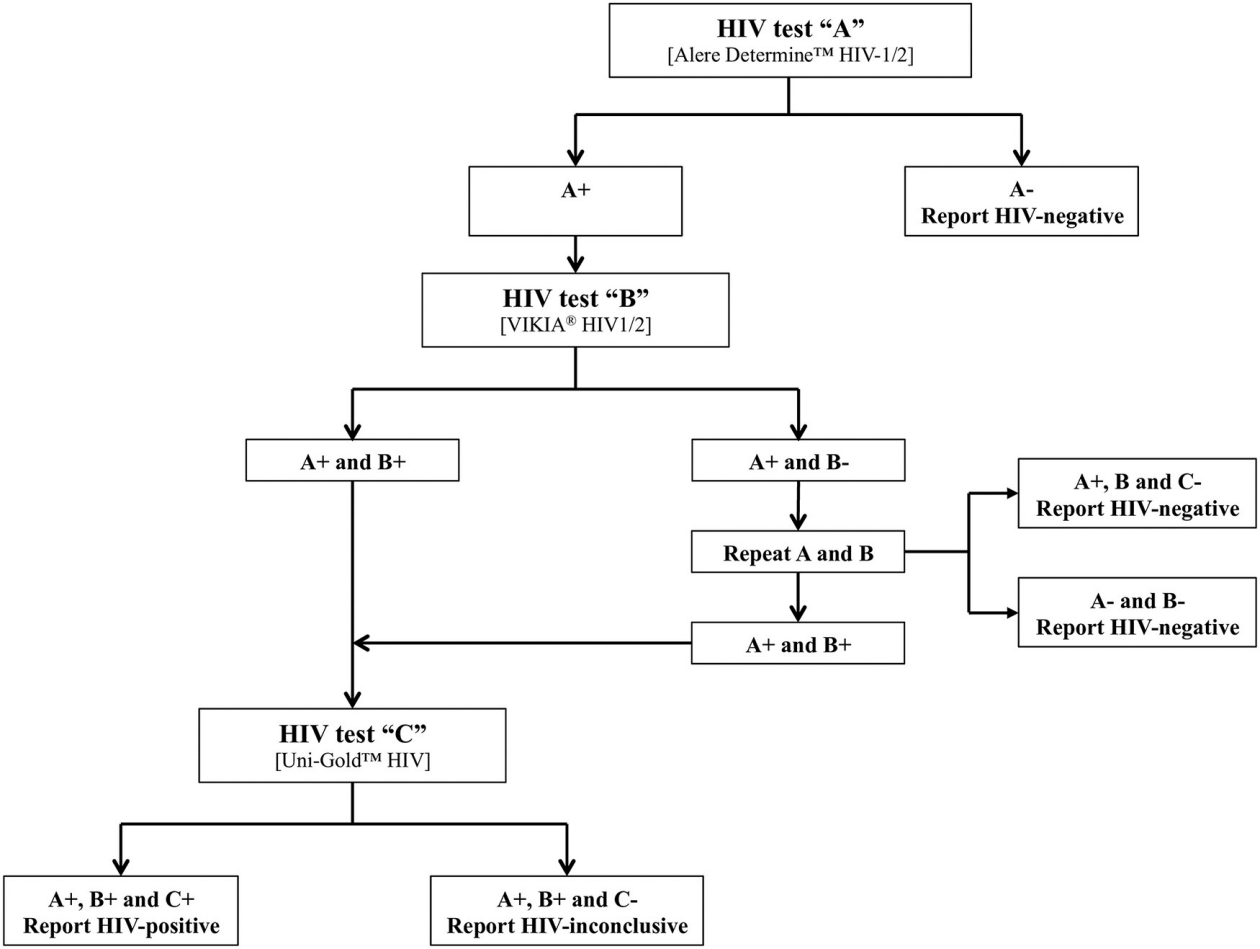
Flow-chart showing the national algorithm of HIV testing in the Democratic Republic of the Congo [12]. Alere Determine HIV-1/2 (Alere, Chiba-ken, Japan), VIKIA HIV1/2 (bioMérieux, Lyon, France), and Uni-Gold HIV (Trinity Biotech, Wicklow, Ireland) were used as the first, second, and third test, respectively.

Five milliliters of blood was drawn by venipuncture by counselors (health care workers) at household visit, only for participants with reactive HIVST following the national algorithm (Fig 1) [12], which is in compliance with the 2015-revised WHO testing strategy [21].

### Ethical statement

The study was conducted after obtaining ethical approval from the Ethics Committee of the School of Public Health of the University of Kinshasa, constituting the National Ethics Committee of the DRC, and from the Ethics Committee of the University of Kisangani. Further permission was obtained from the Tshopo Provincial Health Division. Written informed consent was obtained from all participants or from their parents or guardians if the participants were minors (15 to 17 years) [12]. Anonymity was ensured for all participants. Participants were also informed that they could withdraw from the study at any time without consequences.

### Statistical analysis

Data were entered into an Excel database and analyzed using SPSS 20.0 (Chicago, IL). Proportions were calculated for all categorical variables. A one-sided Wald asymptotic test was used to assess the confidence interval (CI) of difference in acceptability and preference for HIVST before and after self-testing [22]. However, the Wilson score bounds was used for any other estimate of the IC or when the one-side Wald asymptotic test produced outliers [23]. All estimations of CI were at an alpha level of 0.05, corresponding to 95% confidence limits. Categorical data were compared using Pearson’s chi-squared test, while Fisher’s exact test was used when the validity conditions of the latter test were not verified. Mac Nemar’s chi-squared pairing test was used to assess the association between acceptability and preference for HIVST before and after self-testing. The Cohen’s κ coefficient was used to evaluate the concordance between the results read by adolescents and by supervisors.

The sensitivity and specificity of adolescent-interpreted HIV self-test results were calculated, taking into account only the positive and negative test results. Note that, all reactive results confirmed with serologic testing algorithms were considered as a reference for “positive” results. Furthermore, the PPV and NPV were calculated with respect to the reported HIV prevalence in this study using Bayes’ formulae [24].

Finally, multivariate regression was used to identify factors independently associated with main study outcomes. Only statistically significant factors at a *P*-value *≤* 0.05 in the bivariate analysis were entered into the multivariate analysis. Note that, missing data were assigned the null value for conservative estimates.

## Results

### Study population

**Fig 2:**
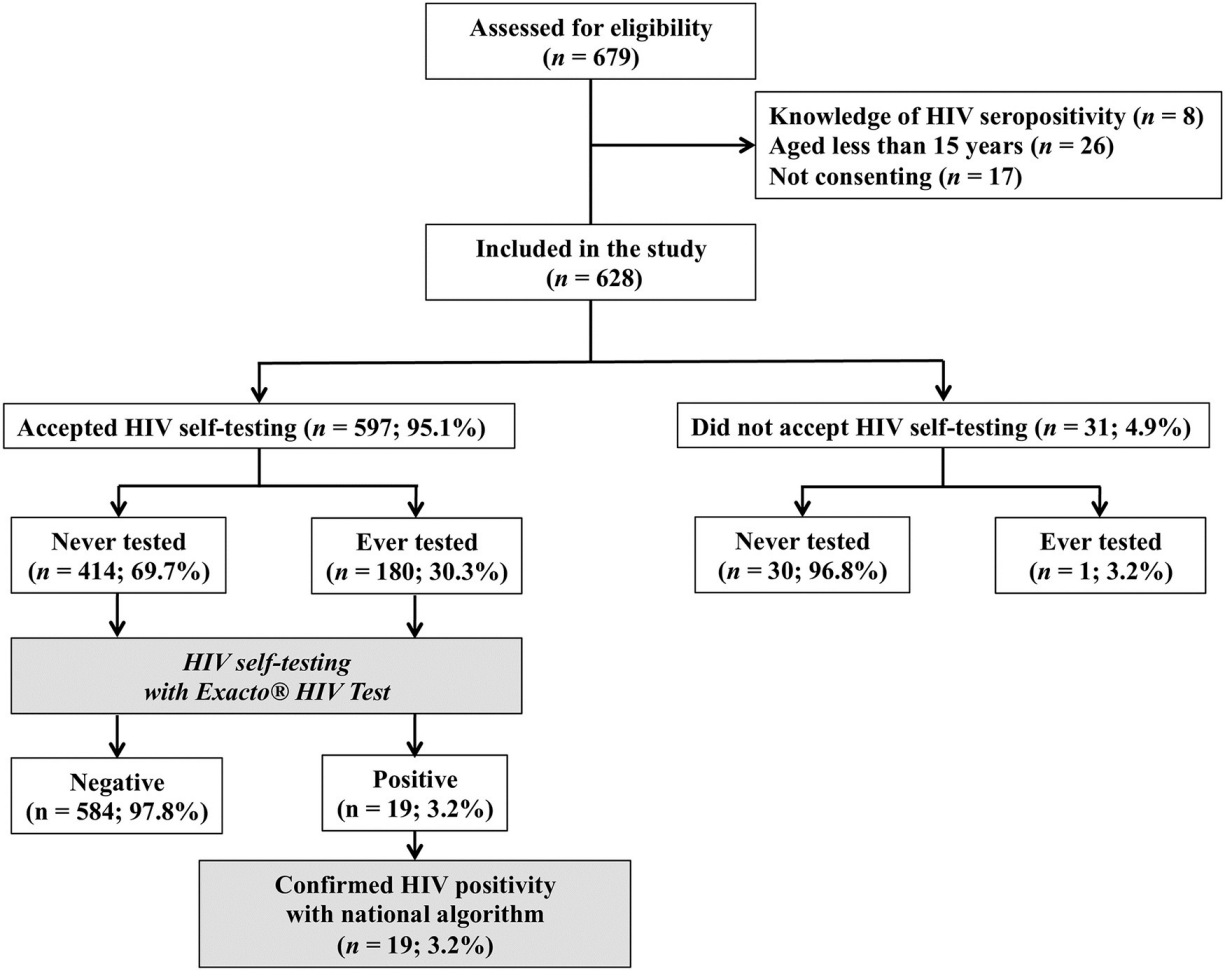
Flow chart showing the recruitment of study participants and their acceptability with regard to using the Exacto Test HIV Self-test (Biosynex, Strasbourg, France).

A total of 353 households were enrolled by the study teams, comprising 679 potential eligible participants. Among them, 628 (83.3%) were successfully enrolled in the study (Fig 2).

Baseline characteristics of study adolescents are shown in Table 1. Among 628 participants (including 369 [58.8%] females), the majority had not yet received HIV counseling and testing. In addition, among participants with a past history of sexual intercourse, majority reported being unaware of the HIV status of their sexual partner.

**Table 1:** Characteristics of 628 adolescents living in Kisangani according to sex.

Characteristics	Sex groups^£^		
	Female [*number* (*%*)]	Male [*number* (*%*)]	Total number* (%)
**Age group**			
15 to 17 years	160 (43.4)	96 (37.1)	256 (40.8)
18 to 19 years	209 (56.6)	163 (62.9)	372 (59.2)
**Partnership and civil status**			
Single	301 (83.1)	238 (94.1)	539 (87.6)
Married/partnered	61 (16.9)	15 (5.9)	76 (12.4)
**Occupation**			
Student	310 (85.2)	253 (97.7)	563 (90.4)
Self-employed^μ^	19 (5.2)	5 (1.9)	24 (3.9)
Unemployed	35 (9.6)	1 (0.4)	36 (5.8)
**Educational level**			
No formal education/ attending primary school	36 (9.8)	19 (7.3)	55 (8.8)
Attending college or technical school	256 (69.6)	145 (56.0)	401 (64.0)
University (currently being attended)	76 (20.7)	95 (36.7)	171 (27.3)
**Number of sex partners in the past six months**			
None	118 (32.2)	62 (24.8)	180 (29.1)
Unique	145 (39.5)	80 (32.0)	225 (36.5)
Multiple	104 (28.3)	108 (43.2)	212 (34.4)
**Sexual orientation**			
Heterosexual	241 (97.6)	176 (93.1)	417 (95.6)
Homosexual	4 (1.6)	7 (3.7)	11 (2.5)
Bisexual	2 (0.8)	6 (3.2)	8 (1.8)
**Condom use**			
Yes, always	61 (25.4)	77 (39.1)	138 (31.6)
Yes, sometimes	67 (27.9)	65 (33.0)	132 (30.2)
No	112 (46.7)	55 (27.9)	167 (38.2)
**Previously tested for HIV**			
Never tested	261 (70.7)	183 (70.7)	444 (70.7)
Ever tested	108 (29.3)	76 (29.3)	184 (29.3)
**Knowledge of the HIV status of sexual partner**			
Yes	12 (9.4)	7 (7.4)	19 (8.6)
No	114 (89.1)	83 (88.3)	197 (88.7)
Not sure	2 (1.6)	4 (4.3)	6 (2.7)
**Previous knowledge about HIV self-testing**			
Yes	58 (15.9)	66 (25.6)	124 (19.9)
No	306 (84.1)	192 (74.4)	498 (80.1)

^£^ Among a total of 628 evaluated participants, 369 (58.8%) were female and 259 (41.2%) were male;

* Missing values were excluded from the Table 1;

^μ^ Self-employed adolescents were engaged in for-profit activities such as trading, motorcycle taxi, carpentry or masonry.

NS: Not significant

### Acceptability

Of the 628 adolescents, 597 (95.1%; 95% CI: 93.1–96.5) accepted the test (Fig 2). The reasons given by these adolescents for accepting HIVST, recommending HIVST, and further distributing HIVST were compared in the pre-testing phase and post-testing phase, as shown in Table 2. A large majority (95.5%) of adolescents declared in the post-testing phase that the easy use of the self-test justified their acceptability, whereas only 67.8% gave this reason in the pre-testing phase, yielding a difference of -27.7% (*P* < 0.0001). Confidentiality concerns were cited by a majority of participants both before and after HIVST (97.4% *versus* 98.5%; *P*: not significant). The rate of acceptability for recommending HIVST to others was higher (96.3%) after HIVST, whereas it was lower (82.5%) before HIVST, giving a difference of -13.8% (*P* = 0.004); difference was of -10% (88.8% *versus* 78.8%; *P* = 0.006) concerning the acceptability to further distribute the self-test.

**Table 2:** Acceptability of and preferences for HIV self-testing according to pre-testing and post-testing phases among 597 adolescents living in Kisangani.

Variable	Pre-testing phase^μ^	Post-testing phase^#^	Difference^£^	*P*-value*
	*n* (%)	*n* (%)	% [95% CI]	
**Reasons to accept HIVST**				
○ Easy to use	419 (67.8)	570 (95.5)	−27.7 [−31.4, −24.3]	< 0.0001
○ Fast results	347 (56.1)	383 (64.2)	−8.1 [−10.6, −6.2]	0.008
○ Confidential	602 (97.4)	588 (98.5)	−1.1 [−2.3, −0.5]	0.254
○ Others**	64 (10.4)	67 (11.2)	−0.8 [−1.9, −0.3]	0.867
**Acceptability of recommending self-testing to other**	515 (82.5)	575 (96.3)	−13.8 [−16.8, −11.3]	0.004
**Acceptability of distributing the self-test to other**	492 (78.8]	530 (88.8)	−10.0 [−12.7, −7.8]	0.006
**Substitution of VCT for HIV self-testing**	344 (55.8)	516 (86.4)	−30.6 [−34.4, −27.0]	< 0.0001
**Willingness to buy HIV self-test**	261 (42.2)	273 (45.7)	−3.6 [−5.4, −2.4]	0.196
**Preference of pre-test counseling**				
○ Not useful	101 (16.4)	274 (45.9)	−29.5 [−33.3, −26.0]	< 0.0001
○ Rather useful	208 (33.8)	141 (23.6)	10.2 [8.0, 12.9]	0.005
○ Useful	170 (27.6)	120 (20.1)	7.5 [5.6, 9.9]	0.011
○ Essential	137 (22.2)	62 (10.4)	11.8 [9.5, 14.6]	0.005
**Preference of post-test counseling**				
○ Not useful	33 (5.4)	9 (1.5)	3.9 [2.6, 5.8]	0.078
○ Rather useful	48 (7.8)	21 (3.5)	4.3 [2.9, 6.2]	0.063
○ Useful	248 (40.3)	161 (27.0)	13.3 [10.8, 16.3]	0.004
○ Essential	286 (46.5)	406 (68.0)	−21.5 [−25.0, −18.4]	0.001
**Preference for different ways of doing counseling**				
○ Face-to-face	482 (78.3)	471 (78.9)	−0.6 [−1.6, 0.2]	0.897
○ Via telephone	120 (19.4)	111 (18.6)	0.8 [0.3, 1.9]	0.886
○ Via internet	14 (2.3)	15 (2.5)	−0.2 [−0.68, 0.28]	0.991

^μ^ Missing values were excluded from the Table 2 in pre-testing phase;

^#^ All of 597 participants assessed in post-testing phase evaluation without missing values;

^£^ Difference assessed with Wald asymptotic test using only data collected from all 597 participants in the post-testing phase paired to those from the pre-testing phase;

* *P*-value calculated using Mac Nemar’s test of paired data;

** Other reasons included such as no stigma and discrimination, and curiosity to use HIV self-testing.

CI: Confidence interval; NS: Not significant; MV: Missing values; VCT: Voluntary counseling and testing.

When determining factors associated with the willingness to accept using the self-test via bivariate analyses, no association could be found.

### Preferences

Individual preferences for HIVST strategy over conventional testing strategy (rate of substitution of voluntary counseling and testing [VCT] for HIVST), for willingness to pay, and for counseling are presented in Table 2. Individual preferences for HIVST strategy over VCT increased after performing the self-test when compared to the phase before HIVST (83.4% *versus* 55.8%; difference: −30.6%; *P* < 0.0001). Willingness to pay for the self-test was low in the present study’s series, without a significant difference before *versus* after performing the test (42.2% *versus* 45.7%; difference: 3.6%) The majority of participants (68.0%) claimed that post-test counseling was essential after HIVST, whereas only 46.5% claimed this before HIVST (difference: −21.5%; *P* = 0.001); face-to-face counselling was greatly preferred over other modes of counseling, without a significant difference between the pre-test and post-test phases (78.3% *versus* 78.9%).

### Feasibility

Overall, among the 597 adolescents who accepted the use of the HIV self-test, 574 (96.1%; 95% CI: 94.2–97.4) correctly used the self-test and succeeded in obtaining an interpretable result, whereas only 23 (3.9%; 95% CI: 2.6 to 5.8) failed. The correct use of the HIV self-test was similarly high in girls (95.2%) and boys (97.6%). Easy and correct identification of the components of the HIVST kit was observed in 94.8% (95% CI: 92.7–96.3).

Almost one-third (34.8%; 95% CI: 31.1–38.7) of the participants carried out the self-test without any help, whereas 65.2% (95% CI: 61.3–68.9) asked for verbal intructions—girls more so than boys (70.7% *versus* 57.3%; *P* = 0.001). Participants requested verbal instructions when interpreting the HIVST results (67.1%) or when using the lancet (42.2%).

A total of 558 (93.5%; 95% CI: 91.2–95.2) adolescents correctly interpreted their self-test results, whereas 39 (6.5%; 4.8–8.8) failed to do so. Misinterpretation of self-test results accounted for 30.4% of invalid tests (all incorrectly interpreted as negative) and 5.8% of negative tests (3.8% incorrectly interpreted as positive and 2.0% incorrectly interpreted as invalid). No positive test was misinterpreted. The Cohen’s κ coefficient between the results read by adolescents and by supervisors was 0.62. The feasibility of HIVST is summarized in Table 3.

**Table 3:** Analytical results of the feasibility to use the finger-stick whole blood self-test (Exacto Test HIV Self-test, Biosynex) and obtain a valid result in a home-based, supervised survey among 597 adolescents living in Kisangani.

A. Feasibility					B. Concordance between adolescents and supervisors for the interpretation of Exacto Test HIV results^£^					
Characteristics	Sex			*P*			Supervisor result^#^			Total
	Female	Male	Total							
	*n* (%)	*n* (%)					Positive	Negative	Invalid	
**Easy identification of the components of the HIVST kit**					**Adolescent result**	Positive	19 (100.0%)	21 (3.8%)	0 (0.0%)	40 (6.7%)
Yes	339 (96.6)	227 (92.3)	566 (94.8)	0.020						
No	12 (3.4)	19 (7.7)	31 (5.2)							
**Successful performance**						Negative	0 (0.0%)	523 (94.2)	7 (30.4%)	530 (88.8%)
Yes	334 (95.2)	240 (97.6)	574 (96.1)	0.133						
No	17 (4.8)	6 (2.4)	23 (3.9)^μ^							
**Need for verbal instructions**						Invalid	0 (0.0%)	11 (2.0%)	16 (69.6%)	27 (5.5%)
Yes	248 (70.7)	141 (57.3)	389 (65.2)*	0.001						
No	103 (29.3)	105 (42.7)	208 (34.8)							
**Correct interpretation of HIVST results**										
Yes	327 (93.2)	231 (93.9)	558 (93.5)	0.475	Total		19 (100%)	555 (100%)	23 (100%)	597 (100%)
No	24 (6.8)	15 (6.1)	39 (6.5)							

^£^ The Cohen’s κ coefficient between the results of reading by participants and the results read by supervisors was 0.62;

^#^ The concordance between peer educator and counsellors (health care worker) for interpretation of HIV self-test results was 100;

^μ^ Misuse of the lancet (15/23, 65.2%) and confusion of blood versus diluent wells on the cassette of self-test (8/23, 35.8%) were the causes of performance failure;

* Most participants have called for verbal instructionseither when interpreting HIVST results [67.1% (261/389)], or when using the lancet [42.2% (164/389)].

HIVST: HIV self-test; n: Number; NS: Not significant; SD: Standard deviation.

Concerning possible factors associated with the feasibility of HIVST, the dependent variables used for this analysis were “successful performance” and “correct interpretation of self-test result.” The following factors were significant in unadjusted bivariate analysis: “age group,” “educational level,” “sexual intercourse in the past six months,” and “knowledge about HIV self-testing’s existence.” Multivariate analysis followed by logistic regression showed that only the variable “educational level” remained associated with the variable “correct interpretation of self-test result” (Table 4): The correct interpretation of HIV self-test results decreased significantly when adolescents had no formal education or attended primary school as compared to those currently attending university (37.0% *versus* 100%; *P* < 0.0001; cOR: 0.01 [95% CI: 0.001–0.02]; aOR: 0.01 [95% CI: 0.001–0.03]). Note that successful performance of HIVST was not associated with any variables in multivariate analysis.

**Table 4:** Bivariate and multivariate regression analysis of factors associated with the successful performance of Exacto Test HIV Self-test (Biosynex) and the correct interpretation of self-test results among the 597 study participants.

Characteristic	Successful performance					Correct interpretation of self-test result				
	*n* (%)	cOR [95% CI]	P*	aOR [95% CI]	P**	*n* (%)	cOR [95% CI]	P*	aOR [95% CI]	P-value**
**Age group** (years)										
15–17	234 (94.7)	Reference	-	NS^£^	NS	225 (91.1)	Reference	-	Reference	-
18–19	340 (97.1)	0.5 [0.2, 1.2]	0.132	NS	NS	333 (95.1)	0.5 [0.3, 1.0]	0.036	0.8 [0.3, 2.1]	0.649
**Educational level**										
No formal education/ attending primary school	48 (88.9)	0.3 [0.1, 0.7]	0.013	0.3 [0.1, 1.5]	0.165	20 (37.0)	0.01 [0.001, 0.02]	< 0.0001	0.01 [0.004, 0.03]	< 0.0001
Attending college or technical school	371 (96.4)	1.2 [0.5, 2.8]	0.695	NS	NS	379 (98.4)	11.8 [4.8, 28.6]	< 0.0001	NA	NA
University (currently being attended)	154 [98.1)	Reference	-	Reference	-	157 (100.0)	Reference	-	Reference	-
**Sexual intercourse in the past six months**										
Yes	410 (97.4)	Reference	-	Reference	-	399 (94.8)	Reference	-	Reference	-
No	155 (92.8)	2.9 [1.2, 6.7]	0.010	0.5 [0.2, 1.2]	0.109	150 (89.8)	2.1 [1.1, 4.0]	0.029	2.0 [0.7, 5.3]	0.179
**Knowledge about HIV self-testing’s existence**										
Yes	119 (100.0)	NA	-	NA	NA	115 (96.6)	Reference	-	NS	NS
No	451 (95.1)	NA	0.005	NA	NA	439 (92.6)	2.3 [0.8, 6.6]	0.113	NS	NS

* *P*-value calculated using Pearson’s χ_2_ test or Fisher’s exact test;

** *P*-value calculated using logistic regression analysis;

^*£*^ NS concerned the variable not included in the logistic regression analysis.

aOR: adjusted Odds ratios; cOR: crude Odds ratios; CI: Confidence interval; NA: Not attributable; NS: Not significant.

### Accuracy

All 19 adolescents confirmed as being HIV positive with the Exacto Test HIV Self-test (Biosynex) were further confirmed as positive with the national HIV testing algorithm (Fig 2), giving an estimated HIV prevalence of 3.2% (95% CI: 2.1–4.9) among adolescents in Kisangani. The sensitivity of adolescent-interpreted HIV self-test results was estimated at 100% (95% CI: 98.8–100.0), its specificity at 96.0% (95% CI: 4.0–97.3), its PPV at 45.2% (95% CI: 41.1–49.3), and its NPV at 100% (95% CI: 98.8–100.0).

## Discussion

In the present study in Kisangani, DRC, HIVST was highly acceptable (≥ 95%) among adolescents aged between 15 and 19 years. Furthermore, the vast majority (≥ 96%) of participants correctly carried out the self-test. Despite frequent (65.2%) verbal instructions, only 93.5% correctly interpreted the self-test results. Finally, adolescents generally (78.9%) preferred face-to-face counseling and mostly (68.0%) post-test counseling. Taken together, these observations demonstrate that adolescents constitute a target population for HIVST with a high acceptability rate, but they also indicate the intrinsic difficulties encountered in terms of both the interpretation of the results and the demand for counseling about the test.

A high acceptability rate for HIVST was observed among study adolescents. High acceptability for the oral fluid-based HIVST were previously reported among adolescents [8, 25–28], attributed to the test’s non-invasiveness, convenience, and ease of specimen collection [19]. Furthermore, high acceptability of blood-based HIVST among adolescents has also been reported in South Africa [7]. These findings imply that the preference for oral *versus* finger-stick self-testing does not constitute a barrier against HIVST that should have been addressed prior to implementing HIVST among adolescents.

The most important factor influencing the acceptability of HIVST is individual motivation to perform HIV testing [19]. Our study showed that the greatest motivation among adolescents to accept using HIVST is the test’s confidentiality, followed by test’s ease of use and fast results. The rate of acceptability to distribute HIVST to others was higher after using the self-test, in keeping with previous observations that showed that secondary distribution of the self-test to other persons could increase the overall acceptability of HIVST [29].

Community-based HIV testing has greatly increased the overall rate of HIV testing in sub-Saharan Africa in the last decade [5,15]. In the present study, door-to-door, community-based HIVST using peer educators was a valuable asset for increasing acceptability of HIVST among adolescents and thus also for breaking down the psychological barriers to voluntary testing as related to stigma, discrimination, and lack of confidentiality [17,18].

The sensitivity of adolescent-interpreted HIV self-test results was estimated at 100%, with a specificity at 96.0%. The WHO recommends a success rate higher than 95% on the major items concerning HIVST practicability, including the interpretation of test results [30]. The difficulty in using a lancet may be challenging, corresponding to decreased acceptability and feasibility of HIVST [7,13]. However, this difficulty was overcome among study adolescents when verbal instructions was provided (42.2% of cases). Several reports evaluating supervised strategies among adults have documented the risk of errors in the interpretation of test results [31–33]. Among adolescents, the risk of error when completing a self-test was less frequent than when interpreting the test [31–33]. The misinterpretation of self-test results concerned mainly invalid tests, as previously reported by Ortblad and colleagues among female sex workers in Uganda [33]. In study participants, the misinterpretation of HIV test results was observed mainly among adolescents with no formal education or who only attended primary school. More generally, a low educational level constitutes the main factor associated with the misinterpretation of HIVST [13,14,34,35].

Simple and easy-to-use devices, instructions for use, and other support tools have been shown to be key to good performance in sub-Saharan Africa, yet may be highly contextual [13,14]. The risk of misinterpretation may be decreased when a brief demonstration is provided before the self-tests are carried out, as previously reported in Malawi [36]. In the present study, a brief demonstration before the provision of self-tests to adolescents was insufficient because the interpretation of self-test results still required verbal instructions among the majority (67.1%) of participants. This latter observation implies that oral assistance is likely essential to the programmatic implementation of blood-based HIVST, as currently recommended by the HIVST policies of the DRC. Mobile phone services, which can operate like hotlines, can use short message service (“SMS”), videos, and phone calls to assist those performing the self-test and to encourage linkage [15].

Concerning preferences, the current study showed that the rate of substitution of VCT for HIVST increased after performing the self-test, although the willingness to pay for the self-test was low. In addition, face-to-face post-test counseling was greatly preferred by the participants. Many previous studies in key and general populations have also reported a preference to have counseling available [17,37–40].

### Strengths and limitations

Our study is original because focusing on the vulnerable adolescent population, who is at high-risk for HIV infection and for whom HIV self-testing could constitute a complementary solution for HIV testing access, in the frequently poorly documented cultural context of French-speaking sub-Saharan Africa. The study has however some limitations. First, the factors associated with acceptability of HIVST were not clearly assessed because a small number (31/628, 4.9%) of study participants did not accept the HIV self-test. Second, selection bias was likely, as the study population comprised mostly women. Indeed, as the dominant emotional responses, fear and anxiety regarding HIV testing were mainly described in females [13]. Finally, providing only blood-based HIVST and supervised HIVST limited the observations in the study to only this type of self-test and distribution approach.

## Conclusions

HIVST is a novel means to make testing more accessible, confidential, and available at non-traditional venues, such as pharmacies and community venues, as well as in the home [6]. The present study demonstrated that (i) the home-based, supervised HIVST using a blood-based self-test and peer-based approach was associated with a high degree of acceptability and feasibility by Congolese adolescents; (ii) the misinterpretation of self-test results constitutes a great challenge to the feasibility of HIVST among adolescents with a poor educational level; and (iii) the face-to-face post-test counselingwas preferred among study adolescents. Future research evaluating the linkage to treatment and care after HIVST will need to complete this study.

## Supporting information

S1 File Raw data of the survey. (XLS)

S2 File Study questionnaire 1 in French (original language). (DOCX)

S3 File Study questionnaire 2 in French (original language). (DOCX)

S4 File Study questionnaire 3 in French (original language). (DOCX)

S5 File Study questionnaire 1 in English. (DOCX)

S6 File Study questionnaire 2 in English. (DOCX)

S7 File Study questionnaire 3 in English. (DOCX)

S8 File Observation sheet in English. (DOC)

S9 File Observation sheet in French (original language). (DOC)

S10 File Standardized sheet of feasibility in French (original language). (TIF)

S11 File Standardised sheet of feasibility in English. (TIF)
