# Supplementary material for: Acceptability, feasibility, and individual preferences of blood-based HIV self-testing in a population-based sample of adolescents in Kisangani, Democratic Republic of the Congo
Source: PLoS One. 2019 Jul 1;14(7):e0218795. doi: 10.1371/journal.pone.0218795 (PMC6602204; doi:10.1371/journal.pone.0218795)
Supplement: S2 File — (DOCX) [file pone.0218795.s002.docx]

**AUTOTEST VIH DIRECTEMENT ASSISTE A DOMICILE CHEZ LES ADOLESCENTS DE KISANGANI EN REPUBLIQUE DEMOCRATIQUE DU CONGO**

**Questionnaire d’enquête**

***A remplir par le participant***

*Le présent questionnaire d’enquête vous garantit l’anonymat et la confidentialité.*

***Questionnaire sur les caractéristiques sociodémographiques***

1. Age :…………....Ans
2. Sexe : Femme  Homme
3. Commune de résidence : Makiso  Tshopo  Mangobo  Kabondo
4. Etat civil : Célibataire  Mariage ou union libre  autre………………………………………………………
5. Occupation : Elève/Etudiant  Avec emploi  Sans emploi
6. Niveau d’étude : Non scolarisé ou Primaire  Secondaire  Universitaire

***Questionnaire sur les risques et comportement sexuels***

1. Avez-vous eu au-moins un rapport sexuel ces six derniers mois ? Oui  Non

1. Si oui, quel est le nombre de vos partenaires sexuels ces six derniers mois : Unique  Multiple
2. Si oui, quel type de rapport sexuel faites-vous ? (i) Hétérosexuel  Homosexuel  Bisexuel
3. Utilisez-vous le préservatif lors du rapport sexuel : Oui, toujours  Oui, par moment  Non, jamais

1. Si vous n’utilisez pas le préservatif lors du rapport sexuel, connaissez-vous le statut sérologique de votre partenaire sexuel (le) ? Oui  Non  Ce n’est pas sur
2. Si oui, le partenaire sexuel avait – il une sérologie positive au VIH ? Oui  Non  je ne sais pas
3. Si oui, le partenaire était – il sous traitement antirétroviraux ? Oui  Non  je ne sais pas
4. Si oui, depuis quand le partenaire sexuel était sous traitement antirétroviraux ? Moins de 6 mois  Plus de six mois

***Questionnaire sur les antécédents de dépistage***

1. Avez-vous déjà réalisé un dépistage volontaire de l’infection à VIH ? Oui  Non
2. Si oui, combien de fois avez-vous réalisé le dépistage volontaire de l’infection à VIH ? 1 fois  2 fois  3 fois et plus
3. Votre dernier dépistage volontaire de l’infection à VIH remonte à quand ? moins de six mois  plus de 6 mois
4. Savez-vous que l’on peut s’auto-dépister à domicile avec des autotests VIH ? Oui  Non
5. Si oui, aviez-vous déjà utilisé un autotest dans le passé ? Oui  Non

*Merci pour votre participation*
