# Supplementary material for: Acceptability, feasibility, and individual preferences of blood-based HIV self-testing in a population-based sample of adolescents in Kisangani, Democratic Republic of the Congo
Source: PLoS One. 2019 Jul 1;14(7):e0218795. doi: 10.1371/journal.pone.0218795 (PMC6602204; doi:10.1371/journal.pone.0218795)
Supplement: S4 File — (DOCX) [file pone.0218795.s004.docx]

**AUTOTEST VIH DIRECTEMENT ASSISTE A DOMICILE CHEZ LES ADOLESCENTS DE KISANGANI EN REPUBLIQUE DEMOCRATIQUE DU CONGO**

**Questionnaire d’enquête**

***A remplir par le participant***

*Le présent questionnaire d’enquête vous garantit l’anonymat et la confidentialité.*

***Questionnaire post-test sur l’acceptabilité et préférence de l’autotest VIH***

1. Quelles sont les raisons vous motivant d’accepter utiliser l’autotest VIH ?

C’est facile à utiliser  L’obtention de résultat est rapide  C’est confidentiel

Autres raisons :…………………………………………………………………………………………………………………

1. Est-il important d’avoir accès à l’autotest VIH ? Oui  Non
2. Accepteriez-vous de recommander l’autotest VIH à une autre personne (ami ou partenaire sexuel) pour utilisation ?

Oui  Non

1. Accepteriez-vous de distribuer l’autotest VIH à une autre personne (ami ou partenaire sexuel) pour utilisation ?

Oui  Non

1. Si l’autotest VIH est disponible, abandonneriez-vous la méthode traditionnelle de dépistage (CDV) à l’hôpital ?

Oui  Non

1. Si l’autotest VIH est disponible, auriez-vous la volonté de l’acheter ?

Oui  Non

1. Et si vous voulez acheter l’autotest VIH, à quel prix achèteriez-vous une pièce d’autotest VIH ? ………………….…$
2. Lors de la méthode traditionnelle de dépistage, le counseling pré-test (avant le dépistage) et post-test (après le dépistage) sont des étapes obligatoires pour une préparation psychique de la personne qui veut faire le dépistage volontaire. Si vous réalisez l’autotest VIH, le bénéfice du counseling vous semble :

- Counseling pré-test : Indispensable  Utile  Assez utile  Inutile
- Counseling post-test : Indispensable  Utile  Assez utile  Inutile

1. Apres avoir réalisé l’autotest, quelles sont vos préférences par rapport aux différentes modalités de réalisation du counseling post-test :

- En présentiel (face à face) : Préférer  Ne pas préférer
- Via téléphone : Préférer  Ne pas préférer
- Via internet : Préférer  Ne pas préférer

*Merci pour votre participation*
