# Supplementary material for: Acceptability, feasibility, and individual preferences of blood-based HIV self-testing in a population-based sample of adolescents in Kisangani, Democratic Republic of the Congo
Source: PLoS One. 2019 Jul 1;14(7):e0218795. doi: 10.1371/journal.pone.0218795 (PMC6602204; doi:10.1371/journal.pone.0218795)
Supplement: S5 File — (DOCX) [file pone.0218795.s005.docx]

**HOME-BASED DIRECTLY ASSISTED HIV SELF-TESTING AMONG ADOLESCENTS IN KISANGANI, THE DEMOCRATIC REPUBLIC OF THE CONGO**

**Survey questionnaire**

***To be completed by participant***

*This survey questionnaire guarantees anonymity and confidentiality.*

***Questionnaire on socio-demographic characteristics***

1. Age :…………....years
2. Sex: Female  Male
3. Residence commune: Makiso  Tshopo  Mangobo  Kabondo
4. Partnership and civil status: Single  Married/partnered
5. Occupation: Student  Self-employed  Unemployed
6. Educational level: No formal education or attending primary school  Attending college or technical school

University (currently being attended)

***Questionnaire on sexual risks and behavior***

1. Have you had at least one sexual intercourse in the last six months? Yes  No

1. If yes, what is the number of your sexual partners in the last six months: Unique  Multiple
2. If yes, what type of sexual intercourse do you do? Heterosexual  Homosexual  Bisexual
3. Do you use the condom during intercourse: Yes, always  Yes, sometimes  No

1. If you do not use a condom during sexual intercourse, do you know the HIV status of your sexual partner?

Yes  No

1. If yes, did the sexual partner have positive HIV serology? Yes  No  Don’t know
2. If yes, was the partner on antiretroviral therapy? Yes  No  Don’t know
3. If yes, since when was the sexual partner on antiretroviral therapy? Less than 6 months  More than 6 months

***Questionnaire on HIV testing history***

1. Have you ever tested for HIV infection? Yes  No
2. If yes, how many times did you self-test for HIV? One time  two times  three times and more
3. Your last HIV testing dates back to when? Less than six months  More than six months
4. Do you know that you can self-test at home with HIV self-tests? Yes  No
5. If yes, did you ever use a self-test in the past? Yes  No

*Thank you for your participation*
