# Supplementary material for: Acceptability, feasibility, and individual preferences of blood-based HIV self-testing in a population-based sample of adolescents in Kisangani, Democratic Republic of the Congo
Source: PLoS One. 2019 Jul 1;14(7):e0218795. doi: 10.1371/journal.pone.0218795 (PMC6602204; doi:10.1371/journal.pone.0218795)
Supplement: S7 File — (DOCX) [file pone.0218795.s007.docx]

**HOME-BASED DIRECTLY ASSISTED HIV SELF-TESTING AMONG ADOLESCENTS IN KISANGANI, THE DEMOCRATIC REPUBLIC OF THE CONGO**

**Survey questionnaire**

***To be completed by participant***

*This survey questionnaire guarantees anonymity and confidentiality.*

***Post-test questionnaire on acceptability and preference of HIV self-testing***

1. What are the reasons for accepting to use HIV self-testing?

Easy to use  Fast results  Confidential  No stigma and discrimination  Curiosity to use HIV self-testing

1. Is it important to have access to HIV self-testing? Yes  No
2. Would you agree to recommend HIV self-testing to another person (friend or sexual partner) for use?

Yes  No

1. Would you agree to distribute the HIV self-test to another person (friend or sexual partner) for use?

Yes  No

1. If HIV self-testing is available, do you abandon the Voluntary Counseling Testing for HIV self-testing?

Yes  No

1. If HIV self-testing is available, would you be willing to buy it?

Yes  No

1. And if you want to buy the HIV self-test, at what price would you buy an HIV test piece? ………………….…USD
2. In the Voluntary Counseling Testing, pre-test (before testing) and post-test counseling (after testing) are mandatory steps for a psychic preparation of the person who wants to do voluntary testing. If you do the HIV self-testing, the benefit of counseling seems to you:

- Pre-test counselling: Not useful  Rather useful  Useful  Essential
- Post-test Counselling : Not useful  Rather useful  Useful  Essential

1. After completing the self-test, what are your preferences regarding the different methods of doing post-test counseling?

- Face-to-face : Prefer  Do not prefer
- Via telephone : Prefer  Do not prefer
- Via internet : Prefer  Do not prefer

*Thank you for your participation*
