# Supplementary material for: Acceptability, feasibility, and individual preferences of blood-based HIV self-testing in a population-based sample of adolescents in Kisangani, Democratic Republic of the Congo
Source: PLoS One. 2019 Jul 1;14(7):e0218795. doi: 10.1371/journal.pone.0218795 (PMC6602204; doi:10.1371/journal.pone.0218795)
Supplement: S8 File — (DOC) [file pone.0218795.s008.DOC]

**HANDLING OBSERVATION SHEET**

**Observation of the handling of the self-test by a adolescent**

*To be completed by the observer.*

*The observer gives the participant an Exacto® HIV Self-Test (Biosynex, Strasbourg, France) box with instructions, the language of which (French, Lingala or Swahili) is chosen by the participant. The observer explains his/her role to the participant, and explains that he/she will play the role of a helpline agent at any time during the handling of the test if the participant requests it.*

| Surname and given name of the observer: ………………………………………………… | | DATE:  ……../……../…………. |
| --- | --- | --- |
| Items |  | Observation |
|  | *Start time of observation* |
| **#1** | **Did the participant recognise the various kit components?** | **YES**  **/ NO** |
| **#2** | **Did he/she wash his/her hands?** | **YES**  **/ NO** |
| **#3** | **Did he/she find the cassette in the sachet?** | **YES**  **/ NO** |
| **#4** | **Did he/she open the diluent vial?** | **YES**  **/ NO** |
| **#5** | **Did he/she disinfect his/her finger correctly?** | **YES**  **/ NO** |
| **#6** | **Did he/she wipe away the traces of alcohol with the compress?** | **YES**  **/ NO** |
| **#7** | **Did he/she use the lancet correctly?** | **YES**  **/ NO** |
| **#8** | **Did he/she form a large drop of blood?** | **YES**  **/ NO** |
| **#9** | **Was he/she able to use the pipette?** | **YES**  **/ NO** |
| **#10** | **Did he/she check that the pipette was filled with blood?** | **YES**  **/ NO** |
| **#11** | **Did he/she deposit the blood into the BLOOD square well?** | **YES**  **/ NO** |
| **#12** | **Did he/she deposit two drops of diluent into the DILUENT round well?** | **YES**  **/ NO** |
| **#13** | **Did he/she start a timer (or equivalent)?** | **YES**  **/ NO** |
|  | *End time of handling* |  |
|  | Did the participant ask for verbal support (telephone call) during the handling? | **YES**  **/ NO** |
| If **YES** at what stage(s) (item number):  **Question(s): Response(s):** | | |
