# Supplementary material for: Acceptability, feasibility, and individual preferences of blood-based HIV self-testing in a population-based sample of adolescents in Kisangani, Democratic Republic of the Congo
Source: PLoS One. 2019 Jul 1;14(7):e0218795. doi: 10.1371/journal.pone.0218795 (PMC6602204; doi:10.1371/journal.pone.0218795)
Supplement: S9 File — (DOC) [file pone.0218795.s009.doc]

**FICHE D’OBSERVATION DE LA MANIPULATION**

*A compléter par l’observateur.*

*L’observateur met à la disposition du participant une boite l’autotest Exacto® Test HIV (Biosynex, Strasbourg, France) avec une notice dont la langue (français, lingala ou swahili) est choisie par le participant. Il explique au participant son rôle et explique qu’il joue le rôle de l’assistance téléphonique à tout moment pendant la manipulation du test, si le participant en fait la demande.*

| Nom et prénom de l’observateur : …………………………………………………….. | | DATE :  ……../……../…………. |
| --- | --- | --- |
| Items |  | Observation |
|  | *Heure de début d’observation* |
| **#1** | **Le participant a-t-il reconnu les différentes composantes du kit ?** | **OUI**  **/ NON** |
| **#2** | **S’est-il lavé les mains ?** | **OUI**  **/ NON** |
| **#3** | **A-t-il trouvé la cassette dans le sachet ?** | **OUI**  **/ NON** |
| **#4** | **A-t-il ouvert le flacon de diluant ?** | **OUI**  **/ NON** |
| **#5** | **S’est-il correctement désinfecté le doigt ?** | **OUI**  **/ NON** |
| **#6** | **A-t-il essuyé les traces d’alcool avec la compresse ?** | **OUI**  **/ NON** |
| **#7** | **A-t-il utilisé correctement l’autopiqueur ?** | **OUI**  **/ NON** |
| **#8** | **A-t-il formé une grosse goutte de sang ?** | **OUI**  **/ NON** |
| **#9** | **A-t-il su utiliser le prélève-goutte ?** | **OUI**  **/ NON** |
| **#10** | **A-t-il vérifié que le prélève-goutte était rempli de sang?** | **OUI**  **/ NON** |
| **#11** | **A-t-il déposé le sang dans le puits carré SANG ?** | **OUI**  **/ NON** |
| **#12** | **A-t-il déposé deux gouttes de diluant dans le puits rond DILUANT ?** | **OUI**  **/ NON** |
| **#13** | **A-t-il enclenché un chronomètre (ou équivalent) ?** | **OUI**  **/ NON** |
|  | *Heure de fin de manipulation* |  |
|  | Le participant a-t-il demandé un soutien oral (appel téléphonique) durant le temps de la manipulation ? | **OUI**  **/ NON** |
| Si **OUI** à quelle(s) étape(s) (numéro de l’item) :  **Question(s) : Réponse(s) :** | | |
