# Supplementary figures and images for: Acceptability, feasibility, and individual preferences of blood-based HIV self-testing in a population-based sample of adolescents in Kisangani, Democratic Republic of the Congo
Source: PLoS One. 2019 Jul 1;14(7):e0218795. doi: 10.1371/journal.pone.0218795 (PMC6602204; doi:10.1371/journal.pone.0218795)

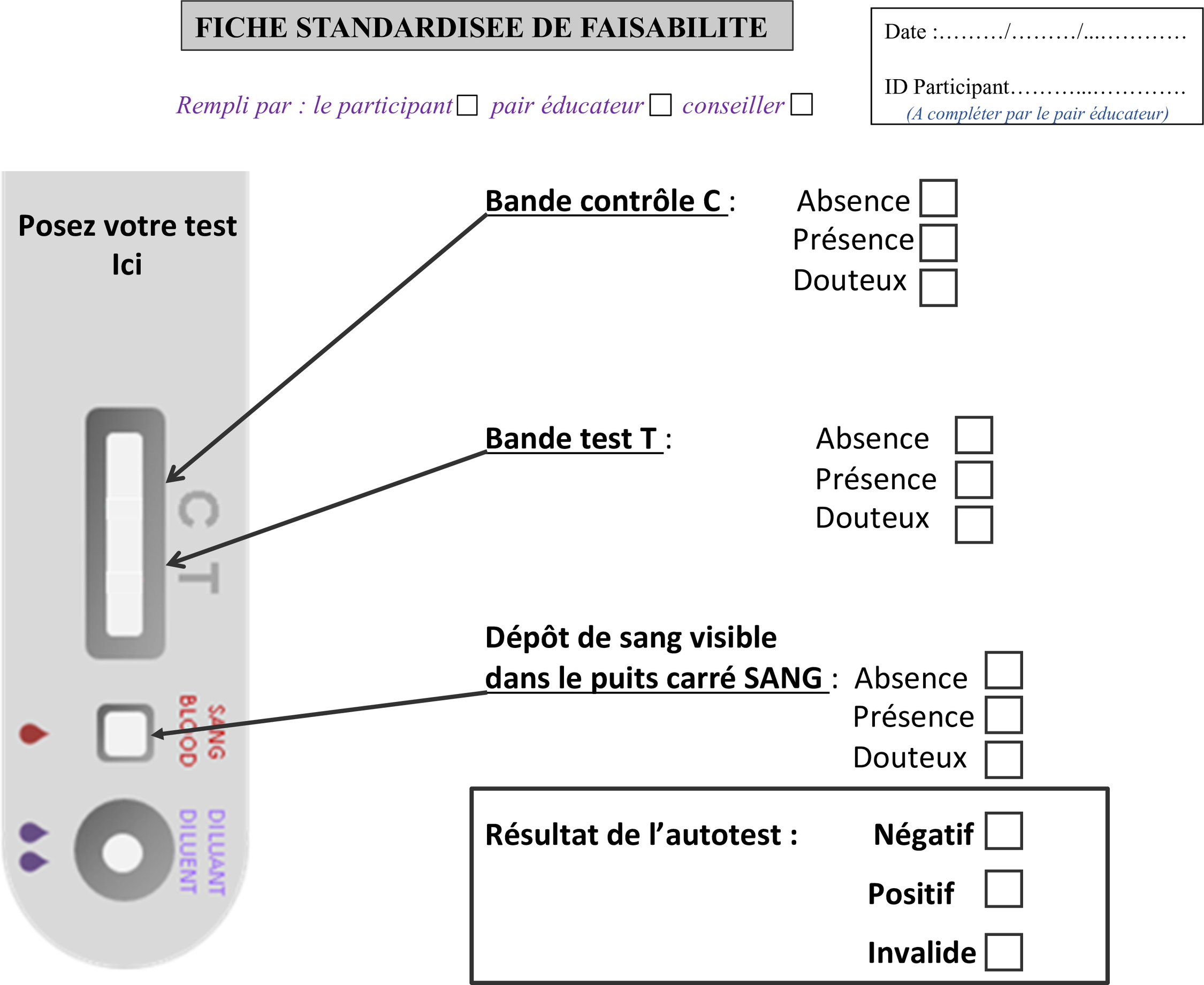

Supplement: S10 File — (TIF) [file pone.0218795.s010.tif]

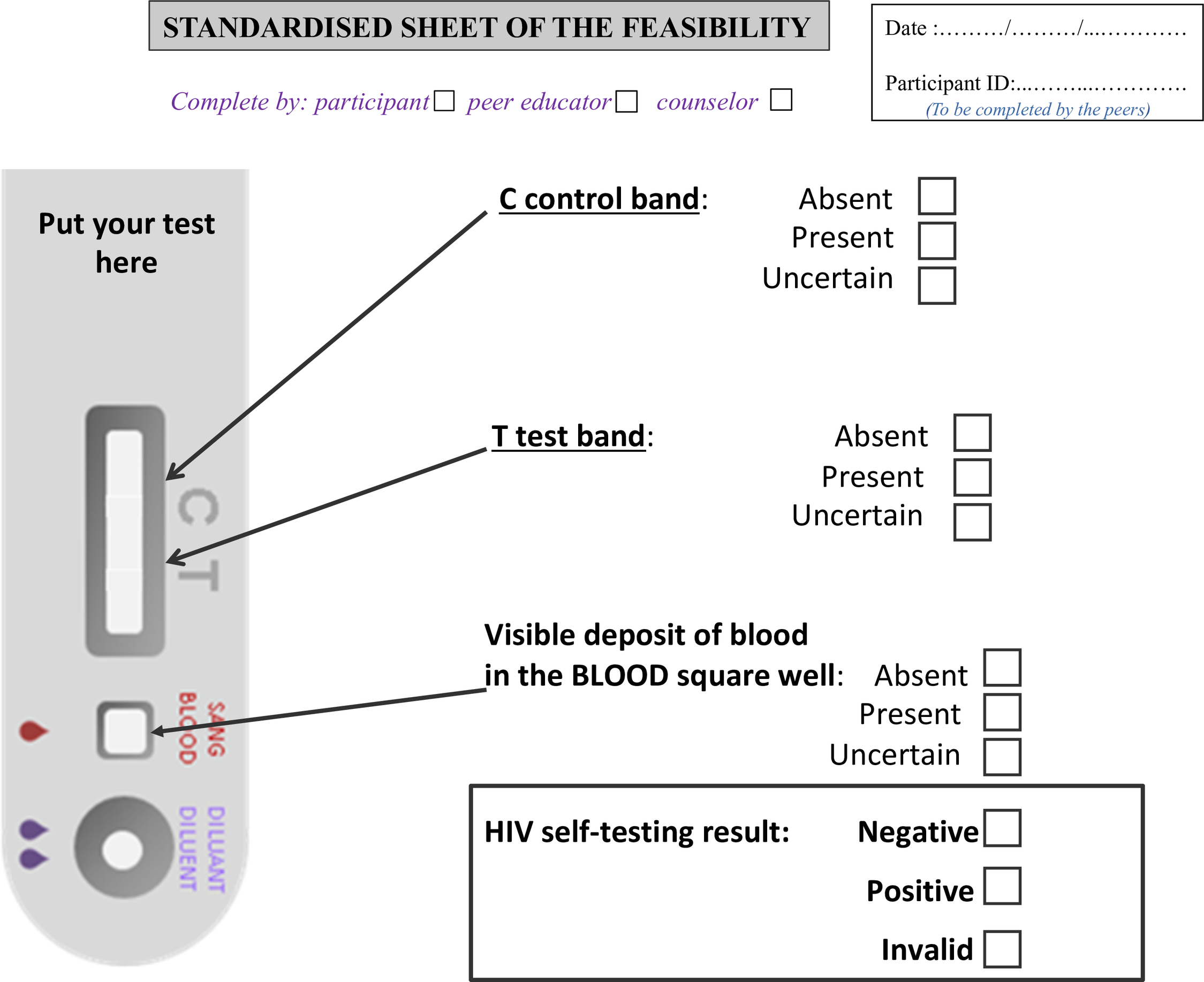

Supplement: S11 File — (TIF) [file pone.0218795.s011.tif]
